# Biological influence of Hakai in cancer: a 10-year review

**DOI:** 10.1007/s10555-012-9348-x

**Published:** 2012-02-19

**Authors:** Luis A. Aparicio, Manuel Valladares, Moisés Blanco, Guillermo Alonso, Angélica Figueroa

**Affiliations:** 1 Servizo de Oncología Médica, Complejo Hospitalario Universitario A Coruña (CHUAC), A Coruña, Spain; 2 Translational Cancer Research Group, Instituto de Investigación Biomédica A Coruña (INIBIC), Complejo Hospitalario Universitario A Coruña, SERGAS, Xubias de Arriba 84, 15006 A Coruña, Spain

**Keywords:** Hakai, E3 ubiquitin-ligase, E-cadherin, Epithelial–mesenchymal transition

## Abstract

In order to metastasize, cancer cells must first detach from the primary tumor, migrate, invade through tissues, and attach to a second site. Hakai was discovered as an E3 ubiquitin-ligase that mediates the posttranslational downregulation of E-cadherin, a major component of adherens junctions in epithelial cells that is characterized as a potent tumor suppressor and is modulated during various processes including epithelial–mesenchymal transition. Recent data have provided evidences for novel biological functional role of Hakai during tumor progression and other diseases. Here, we will review the knowledge that has been accumulated since Hakai discovery 10 years ago and its implication in human cancer disease. We will highlight the different signaling pathways leading to the influence on Hakai and suggest its potential usefulness as therapeutic target for cancer.

## Introduction

Most of the human common tumors are carcinomas; which arise from epithelial cells. Epithelial cells are connected to each other by cell–cell contacts which determine cell polarity and participate in cell differentiation and in the establishment and maintenance of tissue homeostasis. Cell–cell contacts are regulated in epithelial cells during embryonic development and in disease such as tumor development. For instance, the downregulation of cell–cell adhesion is a hallmark characteristic of epithelia–mesenchymal transitions (EMT), a process by which cells lose their polarized epithelial phenotype and concomitantly acquire a migratory or mesenchymal phenotype [1]. E-cadherin is the best characterized and prototype member of the classical cadherins in epithelial cells and is characterized as a potent tumor suppressor, being considered hallmark of tumor malignancy [2]. Epithelial tumors often lose E-cadherin partially or completely as they progress toward malignancy [3, 4]; and most studies have shown its anti-invasive and antimetastatic roles [5, 6]. Given the huge importance of E-cadherin in cancer field, it has been extensively studied the mechanisms involved on its inactivation in human cancers; up to date, it has been addressed several mechanisms: first, a genetic mechanism, such as inherited or somatic mutations; second, epigenetic mechanism, such as hypermethylation of E-cadherin promoter or transcriptional silencing by different repressor including Twist, Snail, and ZEB family members and their respective miRNA regulators; and third, E-cadherin can also be regulated by posttranslational modifications, such as phosphorylation, glycosylation, and proteolytic processing [7, 8]. In 2002, it was identified the first posttranslational regulator of E-cadherin stability, named Hakai [9]; since then many articles have described the emerging biological functions for Hakai protein pointing out its influence on tumor progression and disease. Here, we will review the knowledge that has been accumulated since Hakai discovery 10 years ago.

## Hakai role at adherens junctions

**Fig. 1:**
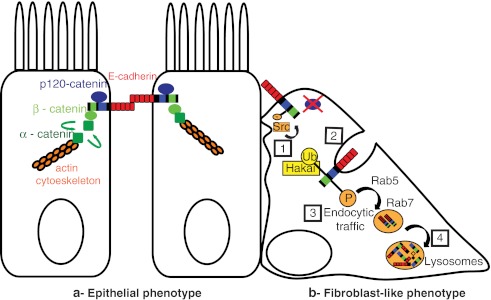
Posttranslational mechanism to donwregulate E-cadherin at AJ by Hakai. **a** Epithelial phenotype. E-cadherin contains five repetitive subdomains to conform the extracellular domain by which it forms homophilic interactions to the extracellular domain of the E-cadherin of the neighboring cell. Its cytoplasmic domain contains two sequences: CH2 domain that interact with p120-catenin and the CH3 domain interacting with β-catenin. It is also linked to the actin cytoeskeleton through the dynamic shuttling of α-catenin between cadherin/β-catenin and actin. **b** Fibroblastic-like phenotype induced by Hakai. After tyrosine phosphorylation by Src in the cytoplasmic domain of E-cadherin, Hakai induces the ubiquitination of the complex, following endocytosis into vesicles through the action of Rab5 and Rab7 to finally degrade into lysosomes, altering the integrity of cell–cell contacts

In polarized epithelia of vertebrates, adhesion between epithelial cells is mediated by distinct junctional complexes named tight junctions, at the apical site, adherens junctions (AJ) at the subapical site, and desmosomes, at basolateral site [10]; these three types of junctions are linked to the cytoskeletal filaments. Although all types of junctions are functionally important, it has been shown that AJ are crucial for the regulation of the dynamics of epithelial cell sheet and can also transmit intracellular signals to the interior of the cell under response of extracellular stimuli [11]. AJ are adhesive structures that are regulated in a Ca^2+^-dependent manner. Calcium-dependent junctions are constitute by a group of type-I transmembrane proteins, and its founding member was termed cadherin [12, 13]. Related molecules identified were also called by several names, such as uvomorulin [14], L-CAM [15], and A-CAM [16]. Original cadherins form a superfamily that is now called “classical” cadherins; however, it has been discovered other members (desmocollin, desmoglein, μ-protocadherin, CNR-cadherin, seven transmembrane cadherin, T-cadherin, and FAT-family cadherins) [17]; moreover, other proteins called nectins were later identified as an AJ components, a family of immunoglobulin-like transmembrane proteins that function in a calcium-independent way to promote cell–cell adhesion [18]. The classical cadherins comprises more than 20 members that contain a common organization domain. The members are called E-cadherin (epithelial, Cdh1), N-cadherin (neuronal, Cdh2), P-cadherin (placental, Cdh3), and so on, each of which shows a distinct tissue distribution [19]. The organization domain consists of an extracellular domain and a cytoplasmic domain. The extracellular domain is divided into five repetitive subdomains, also called cadherin repeats, and every subdomain contain calcium-binding sequences [20]. On its association with calcium, the extracellular domain of cadherins of a cell form homophilic interactions with the extracellular domain of the cadherin of neighboring cell (Fig. 1a, epithelial phenotype). The cytoplasmic domain is highly conserved among classical cadherins and interacts with cytosolic proteins called catenins [21–23]. p120 and β-catenin (homologous to *Drosophila melanogaster* Armadillo) bind to the cytoplasmic tail of the cadherins, and β-catenin binds to α-catenin to form the cadherin–catenin complex. It has been shown that the link of cadherin to the actin cytoskeleton is mediated through the constant shuttling of α-catenin between cadherin/β-catenins and actin which may be a key to explain the dynamic aspect of cell–cell adhesion [24, 25]. Cadherin-based cell–cell contacts are not static but are often dynamically modulated during various physiological and pathological processes including mitosis, epithelial–mesenchymal transition during tumor progression and embryonic development. In all these processes, cadherin has been reported to be downregulated by endocytosis. In epithelial cells, activation of tyrosine kinases such as epidermal growth factor receptor (EGFR), the hepatocyte growth factor (HGF) receptor c-Met, the fibroblast growth factor receptor or Src, induces cell scattering and a fibroblast-like morphology [26, 27]. Met and Src, respectively, have an active function in this process, as they phosphorylate tyrosine residues in the cytoplasmic domain of E-cadherin, thereby promoting its internalization by endocytosis. Fujita et al. [9] underlined the molecular mechanism responsible for this internalization. A new protein named Hakai (which means *destruction* in Japanese) was identified as a responsible for the binding to the tyrosine phosphorylated E-cadherin mediating its internalization and subsequent ubiquitin-dependent degradation thereby altering cell–cell contacts. In the ubiquitination process, ubiquitin moieties itself is involved and three different types of enzymes: the ubiquitin-activating enzyme (E1), the ubiquitin-conjugating enzyme (E2), and a variety of ubiquitin ligases (E3). The E3 ubiquititn ligases provides the specificity as they recognize the substrate through highly specific protein–protein interactions [28]. Hakai, also known as CBLL1, functions as an E3 ubiquitin ligase for E-cadherin that binds to its cytoplasmic domain after tyrosin phosphorylation by tyrosine kinase v-Src. The Src family plays a pivotal role in the regulation of several biological functions associated to changes in morphology, including malignant transformation, cell plasticity, and modulation of intercellular adhesion during EMT [29]. Hakai induces E2-dependent ubiquitination of the E-cadherin complex followed by endocytosis, disrupting cell–cell adhesions and facilitating cell motility under physiological conditions. Although the ubiquitination is one of the most general mechanisms to target cytosolic or nuclear proteins for degradation via proteasome, many membrane proteins have triggered degradation into lysosomes. The first work published in *Saccharomyces cerevisiae* demonstrated that *Ste2p*, a G protein-coupled cell surface receptor, undergone ligand-dependent ubiquitination, following internalization into vesicle to be finally degraded into lysosomes [30]. In fact, it has been shown that upon activation of Src in Madin-Darby canine kidney (MDCK) cells, intracellular E-cadherin is shuttled to the lysosomes for degradation, instead of following normal route of the non-ubiquitinated E-cadherin that is recycled back to the lateral plasma membrane to reform new cell–cell contacts [31–33]. Indeed, the modification of E-cadherin by ubiquitin is essential for its sorting to the lysosomes, which occurs by a process mediated by hepatocyte growth factor receptor substrate and the activation of specific Rab GTPases (Rab5 and Rab7). Rab5-GTP may serve to enhance the rate of E-cadherin transport, and Rab7 activation may serve to shift the balance to endocytic traffic toward the lysosomes. In consequence, cell–cell contacts do not reform and cells remain motile which underlined the first posttranslational mechanism to downregulate E-cadherin during EMT (Fig. 1b, fibroblast-like phenotype) [9–34].

Hakai protein was identified by using yeast 2-hybrid screen of an E10.5 mouse cDNA library (on which a bait vector containing an activated Met-kinase, trp-Met) fused to the C-terminal sequence E-cadherin. The cytoplasmic domain of the E-cadherin contains two sequences, cadherin homology 2 (CH2) and cadherin homology 3 (CH3) domains, conserved between classical cadherins; respectively [35, 36]. p120 and β-catenin can interact with these two domains, respectively; however, its interaction does not depend on the activation of trp-Met. Instead, Hakai interacts with the all cytoplasmic domain, the CH2 domain contains three closed tyrosine residues (in mouse: 756, 757, and 758), of which the first and second ones are specific for E-cadherin and the third one is also conserved between cadherins, including N and OB cadherins. Hakai was unable to interact with these two cadherins, neither with several tyrosine kinase receptors, suggesting that its binding is specific on phosphorylated E-cadherin. By the identification of the crystal structure of p120-catenin in complex with the fragment of cadherin it was proposed that p120 might influence the stability and function in cell–cell adhesion complexes [37, 38]. p120-catenin can interact to the juxtamembrane domain of E-cadherin (including tyrosine-phosphorylation sites, where Hakai is also able to interact), therefore, it was believed that the binding of p120 to the juxtamembrane domain of cadherin block factors such as the ubiquitin-ligase Hakai and components of the endocytic machinery, which tag and target cadherin for destruction and internalization [9, 37–39]. It was proposed that p120 associates with cadherin through both “static” and “dynamic” interactions over an extended stretch of the juxtamembrane domain. The static interactions reflect strong interactions mediated by the highly conserved core of the juxtamembrane domain, and the dynamic interactions, presumably reflect interactions with lower affinity. These motifs in the juxtamembrane domain coincide with those linked to endocytosis by clathrin or Hakai-associated mechanisms [37, 38]. The binding sites for Hakai and p120 are closely apposed in the intracellular juxtamembrane domain of E-cadherin and, accordingly, p120 is displaced by the binding of Hakai to E-cadherin before endocytosis [9]. Moreover, it is well-known that p120 is also phosphorylated by Src kinase and receptor tyrosine kinases [40] and that acidic pH activates c-Src [41]. An acidic extracellular pH is a feature of the tumor microenvironment and has been associated with tumor behavior [42, 43]. Chen et al. showed that acidic extracellular pH induced activation of Src and Fyn and resulting in a tyrosine phosphorylation of E-cadherin and p120 in HepG2 epithelial cells, which was subsequently bound to Hakai and ubiquitin. In consequence, an acidic pH is also weakening the association of E-cadherin and p120 and contributing to the instability of E-cadherin at adherens junctions [41, 44].

## *In vivo* studies of Hakai at adherens junctions

The functional role for Hakai *in vivo* came out in studies carried out in *D. melanogaster*. Hakai is highly conserved in metazoans, however, in *Drosophila*, Hakai did not interact directly to the intracellular domain of *Drosophila* E-cadherin, suggesting not only a different mode of association between these proteins but also that *Drosophila* Hakai does not play a major and direct role in downregulation of E-cadherin levels [45]. As the structure of *Drosophila* Hakai protein predicts cytoplasmic localization, its interaction to *Drosophila* E-cadherin is likely indirect, involving at least components that binds the cytoplasmic Hakai protein and the extracellular or transmembrane domain of E-cadherin. Indeed, Hakai overexpressed was absent from cell–cell interfaces, but it localized with E-cadherin in cytoplasmic vesicles that are different from known endosomal compartments labeled with Rab5, Rab7, or Rab11. These results suggested that Hakai coexist with E-cadherin in an intracellular vesicle compartment that still has not been identified. Only when E-cadherin was coexpressed together with Hakai, they were both found enriched at cell–cell contacts sites. Moreover, by *in situ* hybridization, Hakai mRNA expression was highly detected in blastoderm stage embryos and transcripts persisted up to stage 14; high levels of Hakai mRNA was also detected in migrating endoderm cells, distributed through membranous structure in the cytoplasm, perinuclear region, and the plasma membrane, suggesting its possible implication in the migration of these cells over the visceral mesoderm. As endoderm epithelia and visceral mesoderm do not express E-cadherin, additional *Drosophila* Hakai targets need to be identified and its possible implication in cell adhesion and migration in endoderm and visceral development (for human Hakai substrates see below). In addition, a number of proteins that can interact with *Drosophila* Hakai were identified, underlying the importance of aPKC and TNF-like protein IMD (immune deficiency). In conclusion, that Hakai is essential for early embryonic morphogenesis was demonstrated in *Drosophila*, and may also be involved in regulating multiple target proteins that can influence epithelial development in *Drosophila* [45].

## Hakai molecular structure

**Fig. 2:**
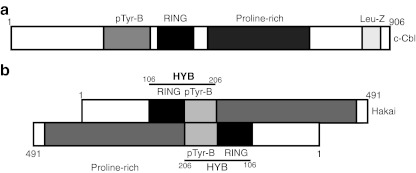
A schematic domain structure of c-Cbl and Hakai proteins. **a** Domain structure of c-Cbl protein contain a phophotyrosin-binding domain (pTyr-B), a RING-finger domain, a proline-rich region, and a lucine zipper (Leu-Z) domain. **b** Molecular structure of E3 ubiquitin-ligase Hakai. A novel domain, HYB (*H*akai pT*y*r-*b*inding) consist of a pair of monomers arranged in an anti-parallel configuration. Each monomer consists of two zinc-finger domains: a RING finger domain and a short pTyr-B domain that incorporates a novel, atypical Zn-finger coordination motif. Both domains are important for dimerization

E3 ubiquitin ligases contain motifs for recognizing specific substrates proteins and are key control points for protein ubiquitination. Up to now, the majority of the ubiquitin ligases identified can be divided into two categories on specific structural motifs: (A) those containing HECT domain and (B) those containing the RING-finger domain [46, 47]. The Cbl family ubiquitin ligase in mammals contains three members: c-Cbl, Cbl-b, and Cbl-c. These members are a single subunit RING-finger E3 containing several motifs surrounding the RING finger: a highly conserved N-terminal phosphor-Tyr binding (pTyr-B) domain that is composed of a four-helix bundle, a calcium-binding domain known as “EF hand”, and an atypical SH2 (*S*rc *h*omology 2) domain. The pTyr-B domain mediates interactions between CBL proteins and the phosphorylated residues on its CBL substrates. The RING finger and pTyr-B domains are separated by an α-helical linker region, important for the regulation on its E3 ligase function. In the C-terminal region, CBL proteins have proline-rich domains that mediate interactions with SH3-containing proteins, and tyrosine that become phosphorylated and mediate interactions with SH2 proteins [28]. In eukaryotic cells, phosphorylation regulates cell signaling by pTyr-B domains typified by the SH2 and pTyr-B (PTB) domains. By molecular modeling, it was assumed that the pTyr-B domain of Hakai was a derivative SH2 domain [9], (Fig. 2a). Hakai contains three domains: a typical RING finger, a short pTyr-B domain, and a proline-rich domain [9], considering that Hakai was structurally and functionally related to c-Cbl. Hakai contains 491 amino acids, sharing 97% homology between human and mouse sequences. The predicted amino acid sequence at its N-terminal contained a C3HC4-type RING finger domain, also present in many E3 ubiquitin ligases [48, 49], and in the C-terminal region, 35% of the amino acids are proline residues. However, Hakai and c-Cbl are not true homologues. A recent study has deepened into the molecular structure of E3 ubiquitin-ligase Hakai highlighting a novel pTyr-B domain [50]. This domain was named HYB (*H*akai pT*y*r-*b*inding) and consists of a homodimer formed at a structurally novel interface. Each monomer consists of two, a RING finger domain and a short pTyr-B domain that incorporates an atypical and novel zinc-finger coordination motif that incorporate connected configuration (Fig. 2b). Therefore, the HYB is constituted by four zinc-binding domains that participate to bind pTyr residues surrounded by acidic amino acids [50]. The previously described target motif was in Src-phophorylated E-cadherin, where two consecutive tyrosine residues (Y753 and Y754 in humans) were reported to be involved in the interaction with Hakai. They analyzed the contribution of these tyr residues and conclude that the phosphorylation of Y754 of the E-cadherin is the only tyrosine significantly involved in the binding, and also that a cluster of negative charges from acidic amino acids around this Y754 of E-cadherin was also important. Indeed, they have shown an important contribution from aspartic acid (D756) and glutamic acid (E757), and significant contributions from valine (V752) and aspartic acid (D750). By mass spectrometry, they also identified a list of novel Hakai-binding proteins phosphorylated by Src. They showed that like E-cadherin, cortactin and DOK1 interact with Hakai only when phosphorylated by Src. Moreover, they suggested that this novel HYB identified was also present in other proteins such as ZNF645, a testis-specific human E3 ubiquitin ligase [51], and possibly in Ligand-of-Numb protein X1 and 2 (LNX1 and LXN2). Hakai and ZNF645 share significant homology, however ZNF645 bound to v-Src phosphorylated E-cadherin but not to cortactin, indicating that they likely have their own sets of targets due to differences found in their sequences between the key zinc-coordinating residues. By tissue distribution, they also suggest that ZNF645, found only in primates, is most likely a recent copy of Hakai and an intronless, indicating that is a retrotransposed copy of Hakai [50]. In conclusion, by combining biochemical and crystal structural analyses, authors demonstrated important mechanism by which E3 ubiquitin ligase Hakai binds its targets molecules. They identified a novel HYB domain that is present in other E3 ubiquitin ligases proteins, often deregulated in cancer and other disease. Therefore, it is suggested that this HYB domain can represent a specific target for directed therapies in cancer.

## Hakai in response to oncogenic signaling pathways

**Fig. 3:**
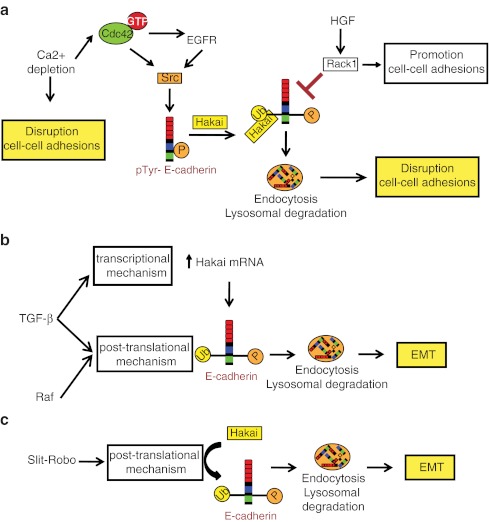
Hakai upstream signaling pathways. **a** Ca^2+^ depletion activate Cdc42 to initiate activation of EGFR and Src, which in turn phosphorylate E-cadherin, leading Hakai-mediated E-cadherin ubiquitination and degradation. The inhibition of Rack1 of the epithelial cell–cell adhesions by regulating Src and growth factor induced endocytosis of E-cadherin. **b** TGFβ and Raf signaling crosstalk to regulate EMT at posttranslational through E-cadherin ubiquitination and degradation. **c** Slit-Robo signaling induces malignant transformation during colorectal cell carcinogenesis through Hakai-mediated E-cadherin degradation

Although it was demonstrated that Src expression regulates ubiquitin-dependent E-cadherin lysosomal degradation [33], it was no until 2008 when for the first time Shen et al. reported upstream regulation [52]. It was previously established that Src, and non-receptor kinase, is a central regulator of signaling downstream of EGFR and it was also shown to regulate EMT by disrupting adherens junctions [53, 54]. On the other hand, Rho GTPases, including Cdc42, Rac1, and RhoA, have all been described to regulate adherens junctions [55, 56]. The majority of previously published data showed that the internalized E-cadherin induced by Ca^2+^ depletion was either recycled back to the plasma membrane or transiently maintained in endosomes of the cells [31, 57]. Given the roles of Cdc42, Src, and EGFR in adherens junction dynamics, it was studied whether they cooperatively contribute to the dissolution of adherens junctions, leading to E-cadherin ubiquitination and degradation. They demonstrated that after Ca^2+^ depletion Cdc42 was activated and in consequence it was initiated the activation of EGFR and Src. Activated Src, in turn, tyrosine phosphorylate E-cadherin, leading to Hakai-mediated E-cadherin ubiquitination. Furthermore, they reported that Cdc42 binds to E-cadherin in a GTP-dependent manner and that this binding is essential for Cdc42 to induce the dissolution of adherens junctions [52] (Fig. 3a).

On the other hand, the role of Rack1 in integrin-mediated cell adhesion is well established; however, the function in cell–cell adhesion through E-cadherin was not clear. RACK1 is one of a group of PKC-interacting proteins collectively called receptors for activated c-kinases (RACKs). It was also previously reported that Rack1 acts as a substrate and inhibitor of Src [58–60], regulating cell growth through the inhibition of Src activity at G1 and mitotic cell-cycle checkpoints and cell survival pathways [61–63]. Taken in consideration these premises, in 2011, it was addressed the effect of Rack1 on Src signaling and its function on E-cadherin-mediated cell–cell adhesions [64]. It was shown that Rack1 promotes cell–cell adhesion by stabilizing E-cadherin and catenins at cell–cell junctions and reduces invasive potential of colon carcinoma cells. Rack1 suppresses the tyrosine phosphorylation of E-cadherin and its binding catenins, attenuating the downstream ubiquitination of E-cadherin, by disrupting Src–E-cadherin–Hakai interactions, and endocytosis of E-cadherin. In consequence, it promotes the reassembly of cell–cell contacts. Moreover, it was demonstrated that Rack1 also block HGF-induced cell scattering by inhibiting E-cadherin endocytosis and junctional disassembly. Therefore, Rack1 targets both Src- and growth-factor-dependent pathways of E-cadherin endocytosis to promote cell–cell adhesion [64], (Fig. 3a).

Given the increasing recognized importance of the EMT process during cancer progression (e.g., from benign adenoma to metastasic carcinoma) several groups have also focused in studying the induction of EMT in response to different signaling pathway and its influence on the E-cadherin downregulation through Hakai action. It is well established that tumor growth factor β (TGFβ) signaling plays an important role in EMT [65]. In fact, adding TGFβ to epithelial cells in culture is a convenient way to induce EMT in various epithelial cells. In several epithelial models, cooperation of the Raf/ERK/MAPK pathways and TGFβ signaling is required for induction and maintenance of EMT *in vitro* and *in vivo* [66, 67]. Janda et al. have demonstrated how MAPK pathway and TGFβ signaling crosstalk to regulate E-cadherin expression at the initial phases of EMT [68]. They have shown that E-cadherin is downregulated at posttranslational level at the onset of the EMT and not at transcriptional level, as it happens to the majority of genes targets regulated through the cooperation between TGFβ and Raf. This is due to an enhanced endocytosis and lysosomal degradation induced by synergistic activation of both pathways. Moreover, they demonstrated that Raf and TGFβ also cooperate in the induction of E-cadherin monoubiquitination; this effect is explained by TGFβ-dependent transcriptional induction of the E3 ubiquitin ligase Hakai and the Raf-dependent tyrosine phosphorylation of E-cadherin. Although Raf cannot directly phosphorylate E-cadherin on tyrosines, an increase in tyrosine phosphorylation upon Raf hyperactivation suggest that Raf/MAPK pathway triggers a multitude of tyrosine kinases, such as Src that may directly phosphorylate E-cadherin (Fig. 3b). However, they suggested that additional mechanism may exist to induce lysosomal degradation and/or proteolytic cleavage of E-cadherin under the cooperative action of Raf and TGFβ as ubiquitin tagging is not sufficient for E-cadherin degradation [68]. Taken all these publications, Hakai seems to be involved downstream of important signaling pathways involved during tumor progression and in consequence more studies are required to elucidate the emerging role for Hakai as a potential therapeutic target.

## Hakai downstream of Slit-Robo signaling

It is well established that the Slits secreted proteins guide neuronal and leukocyte migration through their Roundabout (Robo) transmembrane receptors [69, 70]. Moreover, it has been reported that Slit proteins secreted by solid tumors binds to Robo1 expressed in vascular and lymphatic endothelial cells to stimulate angiogenesis and lymphangiogenesis [71, 72]. Slit2 mediates directional migration of glioma cells [73] and its expression is elevated in human colorectal carcinoma tissues and cell lines [71] and Robo1 was found upregulated in colorectal carcinoma tissues, suggesting the possibility of an autocrine mechanism through which colorectal carcinoma cells secrete Slit2 for signaling through Robo1 expressed on these same cells. Zhou et al. investigated the molecular mechanism of autocrine Slit-Robo signaling to induce malignant transformation during pathogenesis of colorectal epithelial cell carcinoma [74]. They found that the overexpression of Slit2 and Robo1, and also the treatment with ectopic Slit2 of Robo1-positive cells with recombinant Slit1, induced EMT-like phenotype, while knockdown of Robo1 or blockade of Slit2 binding to Robo1 triggered the mesenchymal–epithelial transition (MET)-like phenotype. Robo1-Slit2 recruited Hakai to E-cadherin promoting its ubiquitination and lysosomal degradation. These experimental finding provide evidence for induction of EMT-like phenotype through Hakai-mediated E-cadherin downregulation during epithelial colorectal cell carcinogenesis by autocrine Slit-Robo signaling (Fig. 3c). However, Slit-Robo signaling did not significantly reduce E-cadherin levels through transcriptional suppression of E-cadherin or its known transcriptional repressors (Twist, Snail, Slug, or Zeb2). In this regard, Slit-Robo may resemble TGFβ, HGF, Wnt/Frizzled, estrogen, bone morphogenetic proteins, and microRNAs for reversible epithelial to mesenchymal-like transition and cell migration. These data were consistent to the clinical analysis of 472 clinical cases, where metastasis human colorectal carcinoma tissues samples showed an increase in pan-Slit, mainly Slit2, and Robo1 expression compared to non-metastasic samples. Therefore, these findings also reinforce the possibility to investigate inhibitors of Hakai and/or Slit-Robo signaling in clinical testing for treating colorectal carcinoma.

## Hakai functional role independent of E-cadherin

Other functional roles for Hakai have been addressed independently of E-cadherin protein. This idea came from the novel human Hakai substrates identified [45, 50] and also from the observation of two RNA bands of 2.5 and 4.8 Kb detected by northern blot in most mouse tissues (including testis, heart, brain, spleen, lung, liver, skeletal muscle, and kidney); moreover, mRNA Hakai was detected during developmental stages from days 7 to 17 in postnatal mice. Furthermore, Hakai protein is ubiquitously detected even in tissues that do not contain E-cadherin, such as endoderm epithelia or visceral mesoderm in *Drosophila*, and also in human spleen and skeletal muscle [9, 45]. Indeed, in 2009 Figueroa et al. described polypyrimidine tract-binding protein-associated splicing factor (PSF) as a novel Hakai-interacting protein in cells that do not contain E-cadherin, however they did not obtain evidence showing that Hakai induces ubiquitination of PSF. PSF was firstly identified as an RNA-binding protein, but it was also shown to affect multiple cellular processes, including transcription, pre-mRNA processing, nuclear retention, or DNA relaxation [75–77]. By using cDNA arrays, they determined various specific PSF-associated mRNAs encoding proteins that are involved in several cancer-related processes. Hakai affected the ability of PSF to bind these mRNAs [78, 79]. Furthermore, in this study it is shown that Hakai is involved in the regulation of cell proliferation in an E-cadherin downregulation-independent manner. Hakai overexpression promotes proliferation of various cultured cell lines and the knockdown of Hakai significantly suppressed proliferation of transformed epithelial cells. Additionally, the expression of Hakai was correlated to the proliferation rate in hyperproliferative human tissues Hakai such as endometrium and lymph nodes. Two proteins were proposed to influence proliferation through Hakai: PSF and cyclin D1. They suggested that Hakai may regulate cell proliferation by modulating PSF activity. In Hakai-overexpressing epithelial cells, stable knockdown of PSF partially suppressed the proliferative influence of Hakai overexpression. Moreover, expression of a Hakai mutant lacking the RING finger sequence suppressed cell proliferation, suggesting that this region of Hakai is necessary for promoting cell division. This Hakai mutant was able to bind PSF, indicating that Hakai’s interaction with PSF alone did not promote cell proliferation. The physical and functional interactions between PSF and the E3 ubiquitin-ligase activity of Hakai await further analysis. In another hand, PSF co-localizes with Hakai in the nucleus, raising the possibility that Hakai could play a role in the nucleus through its association with PSF [78, 79]. According to the possible role of Hakai in the nucleus, later report shows that Hakai is a corepressor of estrogen receptor alpha (ERα) in breast cancer cells. By transiently transfection of Hakai, it can repress the transactivation of the ERα through the direct binding to the ERα and by the recruitment of coactivators such as SRC-1 and GRIP-1 (also known as SRC-2) [80]. Hakai overexpression in a tetracycline-induced manner decreased proliferation and migration of ERα-dependent breast cancer MCF-7 cells [80]. These results contrast with previously reported [78] on which overexpression of Hakai increased proliferation in stable cell lines epithelial MDCK. Therefore, Hakai may exert positive or negative control of cell proliferation in different physiological or pathological conditions. Like Hakai, some ubiquitin ligases are reported to function as transcriptional regulators such as E2 ubiquitin ligase BRCA1 that modulate ERα transactivation [81], or RING finger LIM domain-interacting protein that enhanced the activation on targets genes mediated by ERα while it inhibits transcriptional activity of LIM-HD [82]. Therefore, Hakai was proposed as corepressor of ERα playing a role in the development and progression of breast cancer cells. It is possible that the proline-rich domain at the C-terminus of Hakai could be the responsible domain for the repression activity, as this domain is commonly contained in repression domains or near to them, as evidenced in p53 [83], Groucho [84], and HNF4 [85]. As it was previously reported for other E3 ubiquitin ligases [86], these findings suggest that Hakai can have ubiquitin-independent functions in the nucleus or in the cytoplasm that may influence the cell phenotype, in addition to its influence on known substrates (like E-cadherin) or other Hakai interacting proteins.

## Hakai and cancer: clinical applications

There are several lines of evidence that supported a multiple function of Hakai in tumorigenesis. Firstly, it was reported that Hakai induced anchorage-independent cell growth; moreover, Hakai is highly upregulated in human colon and gastric adenocarcinomas compared to normal tissues [78]. New investigations found a low expression of Hakai and E-cadherin or an inverse correlation between both proteins, while comparing several colon adenocarcinomas although the meaning of these results has to be further investigated [87]. Secondly, in epithelial cells expressing E-cadherin shRNA do not extend spiky protrusions that are seen in Hakai-overexpressing cells [88], suggesting that Hakai can also affect cellular phenotypes in an E-cadherin-independent manner. Hakai is localized at the end of the protrusion where FAK, focal adhesion kinase, is also enriched in Hakai-overexpressing MDCK cells, suggesting that Hakai may be involved in regulating the dynamic extension and retraction of these structures and in influencing cell motility [78]. Hakai’s influence on cell attachment to the substrate and invasion capacity of epithelial MDCK cells was addressed [89]. In this system, Hakai overexpression leads to a reduction in cell adhesion to the substrate with impact on decreasing protein levels of Paxillin, a key focal adhesion-associated protein, although its downregulation was controlled by a proteasome-independent mechanism [89]. Nevertheless, further investigations of Hakai in *in vitro* and *in vivo* model systems would lead us to validate its role during tumorigenesis.

Taking together the previous published reports of Hakai during tumor progression, it is increasingly apparent that further investigations of its physiological and pathological functional role would lead us to a novel molecular target for cancer treatment. Up to now, there is only one reported publication on which it is studied the molecular mechanism involving Hakai under the action of an agent against cancer metastasis. One emerging approach in cancer management is the use of “nutraceuticals”, which are relatively nontoxic, cost-effective, and physiologically bioavailable component. Silibinin, a flavonoid from milk thistle seed extract, is a widely consumed dietary supplement that shows a strong efficacy both *in vitro* and *in vivo* against prostate cancer, establishing also its implication on epithelial–mesenchymal transition [90–92]. Despite the previous finding describing the role of silibinin, the main molecular targets responsible for its strong antimigratory and anti-invasive efficacy remained inconclusive. A recent study demonstrated that silibinin inhibits invasive and migratory potential of several highly metastasic cell lines at nontoxic concentrations. Under these conditions, an increased E-cadherin expression at cellular membrane and an inhibition of nuclear β-catenin level was found, accompanied to a decreased level of Hakai, phospho Src (tyr^419^), Slug Snail, and phosphor-Akt (ser^473^) levels, proposing these proteins as a molecular targets implicated in the antimigratory and anti-invasive efficacy of silibinin in prostate cancer cells [93]. These findings have an important translational relevance as the concentration of silibinin used in these studies are within the range of free silibinin levels achieved in the plasma of pancreatic cancer patients in a reported phase I clinical trial [94].

## Other Hakai clinical applications

Apart from the revised functional role of Hakai in cancer, it is also important to mention Hakai importance in other cellular processes and diseases. In a microarray profiling Hakai was found differentially expressed during erythroid differentiation of murine erythroleukemia cells, suggesting the possibility of considering Hakai as a marker for erythropoiesis [95]. Moreover, Hakai was also proposed to be used to evaluate the mechanism of action of immunosuppressive chemicals on which Hakai expression could be novel gene marker for immunosuppression in mouse lymph node assay [96]. More importantly, there are several reported publications that describe Hakai role in infectious disease. The first work describing Hakai in this process was by Krishnan et al. [97]. By using a human genome-wide RNAi screen they identified Hakai as a protein that affect West Nile virus (WNV) infection. WNV is a type of virus known as flavivirus that constitute a significant global human health problem [98]. They identified 20 ubiquitination-related proteins involved and they proposed Hakai to be involved in the cellular internalization of WNV. They demonstrated that Hakai and the proteasome–ubiquitin system are required for the cellular internalization of WNV [97]. This idea was controversial as later report did not support this data suggesting that Hakai is dispensable for cellular internalization during flavivirus entry [99], and that the apparent role of Hakai in WNV infection proposed by Krishnan et al. [97] could reflect an off-target effect produced by the RNA intereference screen used. They rather support a critical role of the ubiquitin/proteasome system during a post-entry step of the WNV life cycle, and that the proteasome activity is required for amplification of several flavivirus genome [99]. Still, the implication of Hakai during WNV infectious needs to be clarified. Other important studies on Hakai in infectious disease were reported in *Listeria monocytogenes*. Upon infection *Listeria* leads to a wide range of symptoms associated to listeriosis, such as gastroenteritis, fetoplacental, and central nervous system infections [100]. Internalization of *L. monocytogenes* with non-phagocytic cells mainly occurs via two bacterial surface proteins: internalin-A (InIA) and internalin-B (InIB) that has E-cadherin and Met as their respective major host-cell surface receptors [101, 102]. InIA interaction with E-cadherin activates β- and α-catenin-mediated signaling pathways involved in the formation of adherens junctions. The initial signals triggered by the interaction of InIA with E-cadherin enhances the internalization of E-cadherin by Src-mediated tyrosine phosphorylation of E-cadherin followed by its ubiquitination by ubiquitin-ligase Hakai [103]. These posttranslational modifications also occur during bacterial infections and are necessary for an efficient InIA-mediated bacterial internalization which it was shown to be dependent on both caveolin and clathrin. All these data document not only how the endocytosis machinery is recruited and involved in the internalization of *L. monocytogenes* but also suggest a functional link between E-cadherin endocytosis and the formation of adherens junctions in epithelial cells [103].

## Conclusions

Taking in consideration the last 10 years of knowledge regarding Hakai, the acquired importance of this protein in cancer and other diseases is increasing apparent. All these reports point out the need to further investigate more deeply the molecular mechanism by which Hakai mediates its action on tumor progression, not only by its influence on EMT though E-cadherin downregulation but also by its influence on early stages of tumor formation. Moreover, novel human substrates in the nucleus or in the cytoplasm for Hakai need to be identified to clarify its influence on cell phenotype, including cell-substrate and invasion capability in tumor cells. Also, more investigations need to address the apparent relevance of Hakai ubiquitin-independent functions that may influence on the cell phenotype. Therefore, Hakai can have different functional roles in different physiological or pathological conditions; in consequence, it is also important to highlight the influence of different signaling pathways on Hakai and to investigate its clinical potential usefulness as therapeutic target for cancer.
